# Development of the Bi-Partite Gal4-UAS System in the African Malaria Mosquito, *Anopheles gambiae*

**DOI:** 10.1371/journal.pone.0031552

**Published:** 2012-02-13

**Authors:** Amy Lynd, Gareth John Lycett

**Affiliations:** Vector Group, Liverpool School of Tropical Medicine, Liverpool, United Kingdom; Johns Hopkins School of Public Health, United States of America

## Abstract

Functional genetic analysis in *Anopheles gambiae* would be greatly improved by the development of a binary expression system, which would allow the more rapid and flexible characterisation of genes influencing disease transmission, including those involved in insecticide resistance, parasite interaction, host and mate seeking behaviour. The Gal4-UAS system, widely used in *Drosophila melanogaster* functional genetics, has been significantly modified to achieve robust application in several different species. Towards this end, previous work generated a series of modified Gal4 constructs that were up to 20 fold more active than the native gene in *An. gambiae* cells. To examine the Gal4-UAS system *in vivo*, transgenic *An. gambiae* driver lines carrying a modified Gal4 gene under the control of the carboxypeptidase promoter, and responder lines carrying UAS regulated luciferase and eYFP reporter genes have been created. Crossing of the Gal4 and UAS lines resulted in progeny that expressed both reporters in the expected midgut specific pattern. Although there was minor variation in reporter gene activity between the different crosses examined, the tissue specific expression pattern was consistent regardless of the genomic location of the transgene cassettes. The results show that the modified Gal4-UAS system can be used to successfully activate expression of transgenes in a robust and tissue specific manner in *Anopheles gambiae*. The midgut driver and dual reporter responder constructs are the first to be developed and tested successfully in transgenic *An. gambiae* and provide the basis for further advancement of the system in this and other insect species.

## Introduction

The major African malaria vector, *An. gambiae*, has attracted considerable scientific focus in the expectation that increased understanding of fundamental mosquito biology may lead to improved tools for vector control [1]. Genome re-sequencing, genetic association studies and high throughput transcriptomic analyses are identifying genes that may have roles in a number of physiological processes related to disease transmission, including innate immunity [2], [3], [4], insecticide resistance [5], [6] and host and mate seeking behaviour [7], [8]. Functional characterisation of these genes through transient RNAi is possible in adult *An. gambiae* in some cases [9], but this approach is limited, not least by the non-systemic nature of gene silencing in mosquitoes [10]. In addition, transgenic technology has been developed in this species [11], [12], [13], [14], [15], but has yet to be exploited extensively to analyse gene function through temporal and spatial mis-expression. To improve the flexibility and utility of functional genomics in *An. gambiae* we are interested in the development of a suitable binary expression system in this species.

The Gal4-UAS system is used routinely and with great success in *Drosophila* and has proven a powerful functional genomics tool. The system is not only used to directly study phenotypes generated through transgene mis- or over-expression, but has a wide variety of applications including enhancer detection and stable gene knockdown through RNAi and refined mosaic analyses [16]. More sophisticated Gal4-UAS tools have recently been developed that permit even finer temporal and inducible control of transgene expression [17], [18], [19].

**Figure 1:**
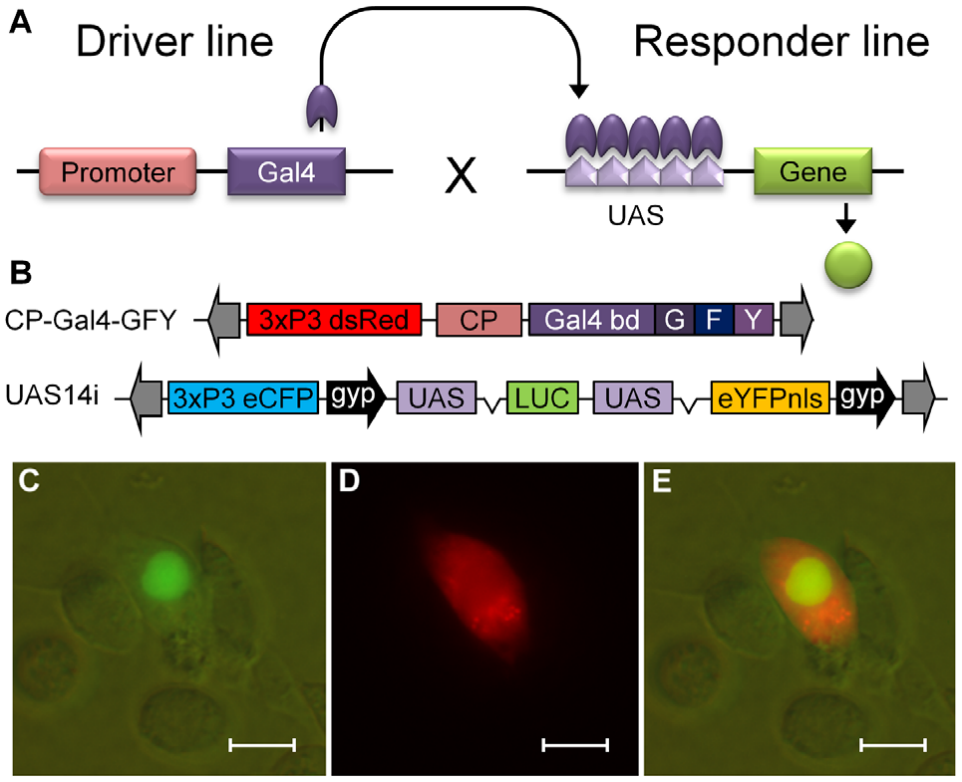
Gal4 driver and UAS responder constructs and eYFP nuclear localization. Illustrations of (A) the bi-partite Gal4-UAS system and (B) the Gal4 driver and UAS responder constructs used for transformation of *An. gambiae* mosquitoes. The driver cassette (upper) consists of the *An. gambiae* CP promoter upstream of the 147aa DNA binding domain from Gal4 fused in frame to three VP16 minimal activation domains, contained within a piggyBac vector carrying the dsRed marker gene under control of the 3×P3 promoter. Grey arrows show piggyBac repeats. The responder cassette (lower) consists of UAS regions upstream of both LUC and eYFPnls, and flanked by gypsy insulator sequences. These are contained within a piggyBac vector marked with eCFP under control of the 3×P3 promoter. (C to E) *An. gambiae* cell line co-transfection with Gal4 driver (pSL*LRIM1-Gal4Δ), responder (pSL*UAS-eYFPnls-g) and a control plasmid expressing cytoplasmic dsRed under the *Drosophila* Actin5C promoter. (C) Localisation of eYFP to the cell nucleus observed with fluorescein filter and bright field (D) Cytoplasmic expression of dsRed observed with a rhodamine filter; (E) A composite image of C and D. Scale bars are 5 µM.

The bi-partite Gal4-UAS approach utilizes transgenic “driver” lines carrying the yeast transactivator, Gal4, under the transcriptional control of a specific regulatory region; and transgenic “responder” lines containing a candidate gene under the transcriptional control of Gal4 binding sites (otherwise known as upstream activation sequences or UAS) [20], [21], [22]. Since Gal4 equivalents are not present in most species, the candidate gene is only expressed in the progeny of crosses between driver and responder lines, when Gal4 and UAS transgenes are brought together in the same genome. The candidate gene is then expressed in the temporal and spatial pattern dictated by the promoter driving Gal4 expression (Figure 1A). Once panels of alternative driver and responder lines are developed, this bi-partite approach allows multiple candidate genes to be expressed in a variety of tissues and developmental stages by simple crossing experiments. This system also allows the analysis of genes whose expression may exert a high fitness cost or dominant lethal/sterile phenotypes, since activation only occurs after crossing. Thus the effects of mis-expression can be studied even if they are somewhat deleterious [23].

The value of the Gal4-UAS system in *Drosophila* has prompted attempts to transfer it directly to other model organisms. However, despite initial reports utilizing the native form of Gal4 in some species, the system was not widely adopted due to low activity of the transactivator and toxicity when over-produced. More recently, various modified forms of Gal4 have been shown to be more active and robust, and have been successfully employed in zebrafish [24], [25], [26], the silkworm [27], the red flour beetle, *Tribolium castaneum* [28] and *Aedes aegypti* [29]. The Gal4 transactivator consists of a DNA binding domain and a transcriptional activation domain that directs mRNA synthesis. Most modifications to Gal4 have involved replacement of the native activation domain with potentially more active, but less toxic, viral or mammalian sequences. Interestingly, the different variant Gal4 transactivators do not behave the same in all species, and it is likely that optimization will be needed to apply this system to other species of interest [28], [30].

Previously it has been demonstrated that the native Gal4 was only minimally active in *An. gambiae* cell culture [30]. To optimize the system for *An. gambiae*, a panel of Gal4 variants were developed, including a series carrying short tandemly repeated synthetic activation domains derived from the Herpes simplex virus protein, VP16, which activates transcription of the intermediate early viral genes. These were assayed in conjunction with an array of responder constructs with different numbers of UAS repeats. This work identified several driver elements that were up to 20 fold more active than the native Gal4, and a responder construct, UAS-14i, consisting of 14 tandemly repeated UAS and a small artificial intron, that allowed the greatest range of activation in *Anopheles* cells [30].

To examine the Gal4-UAS system in *An. gambiae in vivo*, we now report the creation of driver lines containing the Gal4-GFY transactivator [30] under the control of the *An. gambiae* carboxypeptidase (CP) promoter. A CP promoter has been used previously to direct transgene expression in the midgut of adult *Ae. aegypti*, *Anopheles stephensi* and *An. gambiae* mosquitoes [13], [31], [32], [33]. Responder lines carrying UAS-14i regulated yellow fluorescent protein (eYFP) and luciferase genes have also been generated (Figure 1B), and demonstrate for the first time the successful development of a binary expression system in this important disease vector.

## Results

### Development of an insulated dual reporter responder construct

A UAS-14i luciferase construct generated previously for cell line transfection assays [30] was modified for use in transgenic *Anopheles* by the addition of a second UAS14i controlled reporter gene encoding a nuclear localisation signal (nls) tagged eYFP (UAS-eYFPnls). In addition, gypsy insulator elements were used to flank both reporter genes [34]. The function of the UAS-eYFPnls construct was verified initially by co-transfection into *An. gambiae* cells SUA5.1 with the constitutively active LRIM1-Gal4Δ driver plasmid [30] and a control plasmid that expressed cytoplasmic red fluorescent protein, dsRed, from the *Drosophila* Actin5C promoter [35]. The transactivation and localisation of expression was confirmed by eYFP accumulation in the nucleus of the *Anopheles* cells, whilst dsRed was observed throughout the cell (Figure 1 C–E).

### Generation and molecular characterization of transgenic lines

The piggyBac transposons that were used to create the transgenic *An. gambiae* driver and responder lines are illustrated in Figure 1B. The driver (CP-Gal4-GFY) and responder (UAS-LUC-UAS-eYFPnls) cassettes were inserted into piggyBac vectors marked with dsRed and cyan fluorescent protein (eCFP) genes, respectively, under the control of the synthetic 3×P3 eye specific promoters [36].

To produce the driver lines, 274 embryos were injected with the Gal4-GFY transposon, from which 104 larvae (38%) were recovered. Surviving adults (G0s) were separated by sex, placed in six pools and out-crossed to wild type mosquitoes. After screening G1 larvae for dsRed expression, 31 isofemale lines were generated. G2 progeny were scored for fluorescent phenotype, and only those lines that yielded approximately 50% dsRed positive larvae were further maintained. A similar strategy was followed to generate the responder lines. In this case, 188 larvae (20%) were recovered from 942 embryos injected with the dual UAS reporter transposon. G1 larvae were screened for eCFP expression and 41 transgenic isofemale lines were established. Only lines that displayed Mendelian inheritance of the marker gene suggestive of single (or tightly linked) transposon insertions were retained. These lines were assigned a unique one or three letter identifier based on the parental ‘cage’ they were derived from.

Inverse PCR of sequences flanking the transposon insertions was performed on genomic DNA from each isofemale line to substantiate the presence of a single transposon and to identify those originating from different transformation events. From this analysis, six driver and 15 responder lines were identified as unique (Table 1). The precise location of all integrations could not be determined, since three sites reside in highly repetitive DNA that could not be precisely mapped to the genome. In addition, the presence of a second, and thus probably linked, non-canonical integration was detected in the Wlm line. Overall, the minimum rates of *An. gambiae* transformation were approximately one line in 46, and one line in 63 embryoes injected for driver and responder lines, respectively. This is a conservative estimate since several lines that potentially carried more than one insertion were discarded without further characterization. Six of the 21 lines had transposon insertions into predicted genes (VectorBase Agam P3.6), of which one (Xnt) was located in an predicted exon of the orthologue of *dachs* gene, encoding a variant myosin, that is involved in growth of legs and wings [37]. No striking phenotype was observed in this line, although a fitness cost was noted when the line was made homozygous for the insertion. The other genic insertions were within introns of the genes with similarity to *Drosophila Furin2, yata, beat IIIC, and Pbp45* (Table 1).

**Table 1:** Transgene chromosomal insertion sites.

Insert	Line	Chr.	Location	Sequenced region	GenBank accession
Gal4	F		Undetermined	**TTAA**TTATCTATTTTTTTTAATTTGATCTCAATAGTTAGATATAATTCAA	JN585644
				**TTAA**CTGTCCCCACAAAACAATAGTAAGAAGAGCAGTTAATATTTCAGGT	JN585645
Gal4	Dgl	3L-42C	13 Kb 3′ AGAP011375	**TTAA**AAAACAAAACATAGCCAGTAAAAAAATAGGGGACTGTAGGTAAAGA	JN585646
				**TTAA**TTCCGTTAAATAATTTAAAACATCTTCCTCACAATGGACATCTTCA	JN585647
Gal4	Cln		Undetermined	**TTAA**TGAAATTGACATTTATTGGTACATAAATGTGTATGCCATATTGAAT	JN585648
				**TTAA**TATAAATGGTTTCCATTGCATTAAGACACTTATATTTTGATTTACG	JN585649
Gal4	Drt	3R-34A	40 kb 5′ AGAP009328	**TTAA**CCACAAAGCGATC	
				**TTAA**AATTTTATTGTACATCCTAACGTTTGCGAACCATAACTCAAAACCA	JN585650
Gal4	Ivr	UN	AAAB01008832-1	**TTAA**ATACATATTTTAAGGTATCTCCTTCCACTATCCGCAAAATAGTGCC	JN585651
				**TTAA**ATAAATTGTGCTTTGTGTCGATATGTTTTGTTAAACTTAACGCACA	JN585652
Gal4	G	3L-41A	1 kb 3′ AGAP010982	**TTAA**GCAGCTAATGTTGACATCGAATGAAAATGGGTTGAGAATATGAATG	
				**TTAA**GCAATTTTAGCTATTTGGGAGGAATATTTAGTGATTTGGTGTGATT	JN585653
UAS	Mvs	X-6C	11^th^ Intron AGAP001091	**TTAA**CGCTGTTACCGCAAGAGCTTGAAGCAAGAAGCGTTTCACACCATCA	JN585654
			Orth ***Dmel\Yata***	**TTAA**AGGCAACAACAACAACAGAAAATAACGCATCGCGTAGAAACAAACA	JN585655
UAS	Mbl	3R-29C	2^nd^ Intron AGAP007933	**TTAA**CGATGATC	
			Orth ***Dmel\BeatIIIa/b***	**TTAA**TGGGCTGATGTGTGTGTGTGTATGTCCACAGTGCTACCAGAGTCGG	
UAS	Mdy	UN	AAAB010008887-1	**TTAA**AGAAAAACAGCAGTTAGTACGATC	
			22 kb 5′ AGAP012481	**TTAA**GGAAAACGTATTTTTTTTTAATTTATGAATTGCTCGGCCACGTTGG	
UAS	Wnd	2R-18C	58 kb 5′ AGAP004230	**TTAA**CATCTTTAACATGCTGTTTTACCACTGATTTCGCATGATTTTGTTG	
				**TTAA**AGATGATAATATACAGTGGCGGCCACCTACAGTCAGACACCTCACC	
UAS	Tgr		Undetermined	**TTAA**AAAAATCGCCGGTAGCTGGAGAAGCAGCAGAGGTTATGTTTTTTCC	JN585656
				**TTAA**ACGACAGTTGCTTTGGAGTTTGTGAAAAATTTGTAAAAATTATAGA	JN585657
UAS	Nbt	3L-38B	2^nd^ Intron AGAP010319	**TTAA**TGATATAATTAAATGTGTCTGAAATTATAAATACTAATTTTCGTTT	JN585658
			Orth ***Dmel\Pbp45***	**TTAA**TGTCGTTTAAATCAAACATAAAAGCATAACAGGTTGGCTGATAAGT	JN585659
UAS	Wby	2R-13C	1^st^ Intron AGAP002915	**TTAA**CTACCAAGATTTTTTTTTTATTTTTTGCTCAATAGCTTTACTTTTC	JN585660
			Orth ***Dmel\Fur2***	**TTAA**GAGTAGACGTTAAATCAGGGTGTTGTTGAGAAGATTGATGTTTCCA	
UAS	Wlm*	3R-34B	7 kb 3′AGAP009464**	**TTAA**GCGGGAAGAGGACGGTTTGCATGATC	
				**TTAA**ACCGCACTCCAGAAGTGATGAGTTATGAGGGGATGAGAATCGTCCT	JN585661
UAS	Xnt	3R-36B	11^th^ Exon AGAP009933	**TTAA**GGCTGCCATTGACTTCGAACCCGCGTTTCCGCTAATCGTGGACGCA	JN585662
			Orth ***Dmel\d (dachs)***	**TTAA**ACAATGCTGTTCGGCATGCAACAGATGGATTTATTGGTAAGAATGG	JN585663

The table shows type of transgene cassette (Gal4 or UAS); the name of the line; the chromosomal integration site (Chr) into the genome (if known) by chromosome name and band (UN indicates that the region is located on an unknown chromosome); the proximity to nearest predicted gene in VectorBase, the *D. melanogaster* orthologue in intragenic insertions, and the contig number of unknown chromosome insertion sites (location); the sequence obtained by inverse PCR in both directions from the insertion site (sequenced region); and GenBank accession numbers of flanking sequences longer than the submission threshold of 200 bp (GenBank). Of the five insertions into annotated genes, four (Mvs, Nbt, Wby and Xnt) are 1∶1 orthologues of *D. melanogaster* genes and one (Mbl) is part of an orthologous group as predicted in VectorBase.

*Line Wlm also displays salivary gland CFP expression. It also contains two transposons, one canonical.

**and one containing piggyBac sequences indicative of a non-TTAA dependant insertion (not shown).

### Expression and differentiation of fluorescent marker genes

Using appropriate filter sets, larvae and pupae carrying the driver and responder constructs were readily identified by their red or cyan fluorescence, respectively, using low magnification stereomicroscopy. The eCFP fluorescence in different lines was generally less intense than dsRed, as expected from their relative molar extinction coefficients and quantum yields [38]. However, the spectral separation of the fluorescent proteins allowed progeny carrying both markers to be easily distinguished (Figure S1), with no discernable bleed-through between dsRed and CFP filters.

In the majority of the transgenic lines generated, expression of the fluorescent marker proteins in larvae was not limited to the Bolwig organ in the eyes, but also seen in central nervous system ganglia and anal papillae. In two lines, expression was also noted in the larval salivary glands, as described previously [39]. One UAS line in particular, Wlm, gave bright salivary gland expression of eCFP in larval and adult stages (Figure S2), which allows simplified dissection of this tissue. Only one line, Ivr, showed inconsistent expression of the fluorescent transformation marker between individuals. Expression in this line showed a range of phenotypes between strong expression in the eye and central nervous system ganglia to weak expression in one eye only. Consistency in expression did not respond to selection suggesting a non-heritable component, and is most probably caused by position effect variegation [40]. This conclusion is supported by transgene localization amongst highly repetitive sequences (Table 1).

### Midgut specific expression of Gal4 dependent reporter genes

**Figure 2:**
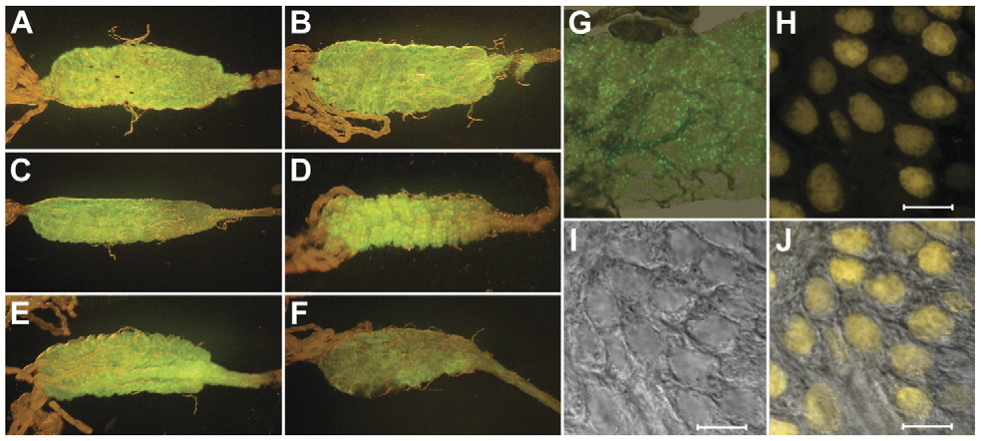
Expression of fluorescent protein in the midgut. Images A to F show a representative image of eYFP expression in the female midgut of driver lines Cln, Drt, Dgl, F, G and Ivr respectively crossed with the responder line Mbl, photographed through a GFP-B filter. (G) Midgut eYFP expression at 100× magnification photographed through a fluorescein filter. Images H to J are confocal microscope images taken with YFP filtering (H), transmitted light (I), and merged (I) demonstrating localization to midgut nuclei. All guts are from sugarfed mosquitoes Scale bar is 10 µm.

To initially examine Gal4-UAS system functionality, six UAS lines (Nbt, Mvs, Mbl, Tgr, Wnd, and Mdy) were crossed with two Gal4 driver lines (F and Dgl). From all crosses at least 10 female progeny were dissected to examine eYFP expression. In all progeny examined, eYFP was detectable in adult female midguts, when viewed through a stereomicroscope (×40 magnification) and in most cases the signal was sufficiently robust to be visible externally through the abdomen cuticle prior to dissection. As expected, the eYFP reporter gene was effectively targeted to the nuclei of midgut epithelial cells (Figure 2 H–J), and was not detected in any other tissues. Preliminary luciferase assays also demonstrated midgut specific expression in all crosses examined (data not shown). Furthermore, eYFP was not detected in any tissue, including the midgut, in any of the non-crossed UAS lines examined. Two responder lines, Mbl and Wnd, chosen for their greater general fitness during colony maintenance, were then used in further assays.

All six Gal4 driver lines were then crossed with Mbl and Wnd UAS lines. Again, all the resulting progeny displayed adult female midgut specific eYFP expression in the non-blood fed state, although the intensity and distribution of eYFP appeared to vary to some extent depending on the driver used (representative individuals shown in Figure 2 and S3). eYFP expression occurred throughout midgut, but in the majority of crosses was more highly expressed in the posterior end. In the most intensely fluorescent midguts eYFP was also visible in the foregut. Only progeny from crosses with line Ivr, that displayed position effect variegation of the eye marker, showed discernable variation in eYFP expression in the midgut between individuals.

**Figure 3:**
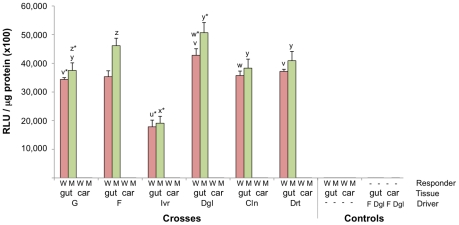
Luciferase activity in midguts and carcasses of female mosquitoes. Mean luciferase activity from dissected midguts and remaining carcass is shown for progeny heterozygous for Gal4 and UAS cassettes (RLU = Relative Light Units, N = 6). All samples are from sugarfed mosquitoes. Controls for both responder lines are homozygous for UAS cassette (N = 3), and the control driver lines F and Dgl are homozygous for Gal4 cassette (N = 6). The responder line (W = Wnd, M = Mbl), the tissue examined (gut = midgut, car = carcass) and the driver line utilized in each cross and in the controls, are indicated along the X-axis. Error bars show standard errors. Significance differences (Mann Whitney, p<0.05) in midgut specific expression between crosses involving different driver lines but the same responder line are indicated by letters above each bar. u* and x* indicates significant difference between progeny from these crosses compared to all others. Other letters marked * (ie v*, w*, y*, z*) indicate significant difference only to those crosses bearing the same letter (ie v* is significantly different to v).

To quantitate Gal4 mediated UAS activation, luciferase assays were performed on dissected non-bloodfed midguts and the remaining carcass of adult progeny from all crosses of the six driver and two responder lines. Overall, specific luciferase activity in the midgut was over 50,000 times greater in all of the alternative transheterozygous (Gal4-UAS) progenies than in the respective homozygous UAS lines (Figure 3). Apart from the Ivr line that displayed positional effect variegation, the level of luciferase activity was consistent between all driver and responder line crosses, varying less than 1.4 fold between the lowest (G) and highest (Dgl) expressing lines. Although there were significant differences between some combinations of driver and responder crosses (as indicated in Figure 3), only midguts derived from Ivr line driver crosses had significantly different activity (Mann Whitney, p<0.01) than all other driver line crosses with both responder lines.

In agreement with the absence of background expression observed with the fluorescent reporter, the more sensitive luciferase assay detected only a very low level of activity in carcasses taken from all crosses (Figure 3). In addition, luciferase activities in midguts from the non-crossed UAS responder lines were similar to baseline readings with mosquito extract-free blank controls. The maximum amount of Gal4-dependent luciferase activity observed in the carcass was only two fold greater than found from non-crossed UAS controls.

### Sex-specific expression of reporter genes

**Figure 4:**
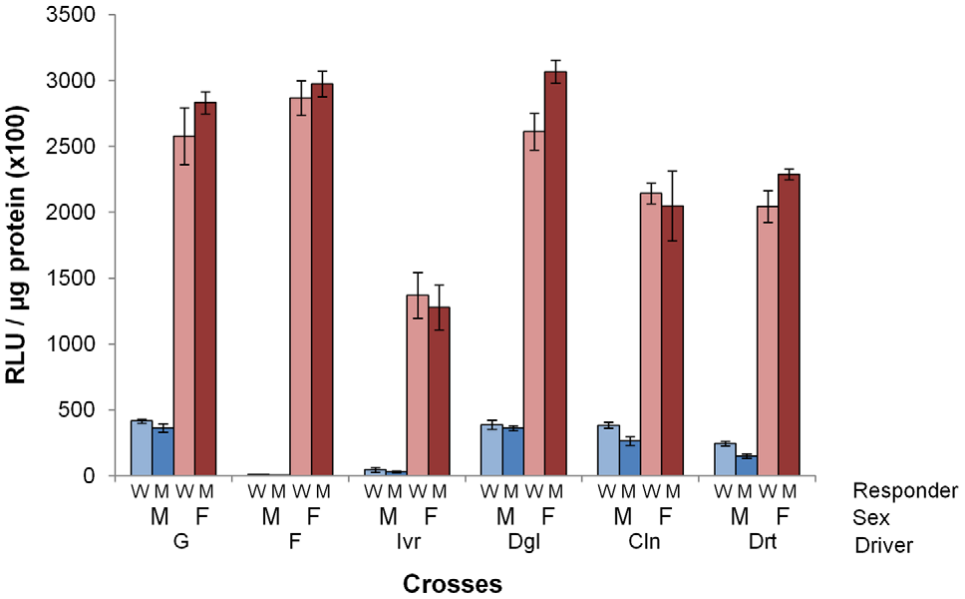
Luciferase activity in male and female mosquitoes. Mean luciferase activity in whole male and female sugarfed mosquitoes for all crosses between six driver lines and two responder lines (RLU = Relative Light Units). Driver lines, responder lines and sex are indicated along the X axis. All mosquitoes were heterozygous for Gal4 and UAS cassettes (N = 6). Error bars show standard errors. There are significant differences between male and female luciferase activities in all crosses (Mann Whitney, p<0.01). Luciferase activities in males from crosses involving F and Ivr driver lines are significantly different to those males from all other driver line crosses (Mann Whitney, p<0.05).

Whilst eYFP was readily observed in female midguts after dissection, fluorescence in male midguts was apparent, but less intense in progeny from all crosses, except for those derived from driver line F, in which eYFP was not detectable. (Figure S4B). Similarly, luciferase assays of male and female mosquitoes also demonstrated significantly enriched female specific expression in all Gal4-UAS crosses (Mann Whitney, p<0.01) (Figure 4). In addition, differences in the magnitude of male luciferase expression were observed depending on the driver line used in the cross, which correlated well with observed intensity of eYFP fluorescence. For example, the F driver gave the most female specific expression, which was over 300 fold greater than male, whereas in the strongly fluorescent male guts observed from the Dgl driver line crosses (Figure S4A) the relative difference in luciferase activity was nine fold between sexes (Figure 4).

### Response to blood feeding

**Figure 5:**
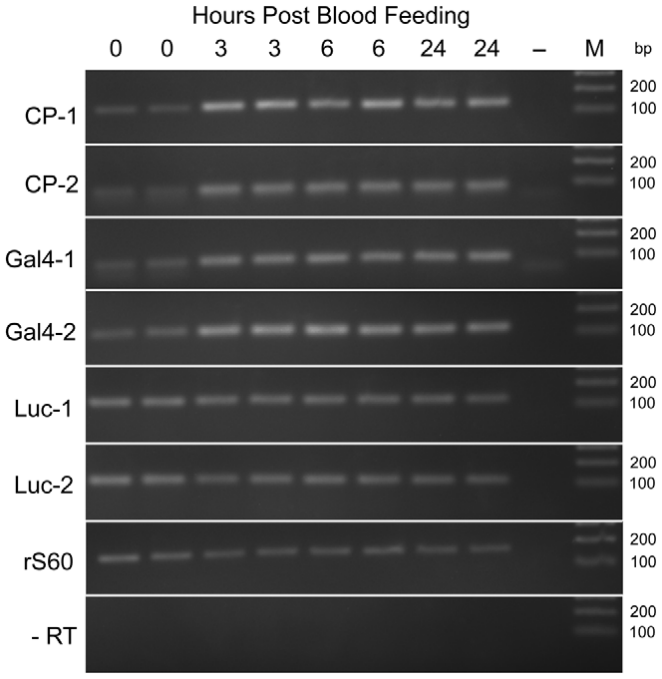
Semi-quantitative RT-PCR of midgut transgene transcription in response to blood feeding. RT-PCR of RNA from female mosquito midguts heterozygous for the Gal4 and UAS transgene cassettes. PCR reactions from two RNA preparations are shown for each timepoint after bloodfeeding. Results from unfed (0 hour), 3, 6 and 24 hour timepoints are shown plus a cDNA free negative control (−). A DNA ladder is shown with sizes indicated (M). Two independent amplicons were amplified for the native carboxypeptidase gene (CP), the Gal4 transgene (Gal4) and the luciferase transgene (LUC). Ribosomal S60 (rS60) was used to standardize for RNA quantity and a DNA contamination control was performed using rS60 primers (-RT).

No obvious variation in eYFP expression in the midgut or other tissues was noted in all transheterozygous individuals examined following ingestion of a bloodmeal. Furthermore, luciferase assays also suggested that activity was not upregulated throughout 24 hours post-bloodfeed (data not shown). This absence of response was then examined at the level of transcription, through semi-quantitative RT-PCR on midgut RNA isolated before and at different time points following a bloodmeal. As is shown in Figure 5, the Gal4 gene follows a similar temporal pattern of upregulation as the endogenous CP gene, whereas luciferase appears to be constitutively expressed prior to blood feeding and no discernable change in luciferase transcription was detected during the 24 hours examined.

## Discussion

The modified Gal4-UAS system utilized here worked efficiently in *An. gambiae* to give robust expression patterns. Crossing of driver and responder lines resulted in successful transactivation of the luciferase and eYFP reporter genes in the midgut epithelia of resultant progeny. This expression was observed in all individuals characterised from all 20 different crosses carried out, involving six different driver lines and six UAS lines. The phenotypes were stable over time and consistent expression was observed in transheterozygous progeny after the driver and responder lines had been maintained for at least 25 and 20 generations respectively.

As the first responder lines developed for *An. gambiae*, we have utilised a construct carrying both UAS regulated eYFPnls and luciferase. These lines permit fluorescence-based identification of temporal and spatial expression patterns, whilst luciferase activity measurements allow simple and accurate quantification. The nuclear tagged fluorescent protein was chosen to allow differentiation of reporter gene expression from cytoplasmic directed expression of marker genes should future experiments require crossing with lines carrying fluorescent proteins with overlapping emission spectra.

Gypsy insulator sequences were placed around the UAS cassettes to potentially mitigate position effects of transgene insertion [41], and are expected to boost transgene expression in all tissues by preventing the repressive action of nearby silencers and chromatin structure. In *Drosophila*, it has been shown that the different sites of transposon integration do not support the expression of transgenes equally well in all tissues, and that this can be largely overcome by the insulation of inserted genes with gypsy sequences [42]. The robust expression of eYFP and luciferase in all progeny examined suggests that the gypsy elements may also function to equalise transgene expression between transgenic lines. Although this will be tested more rigorously as further driver and responder lines are developed. The consistency of midgut expression from all six of the driver lines contrasts markedly with recent production of nine transgenic *Ae. aegypti* lines without insulator elements, of which only two robustly expressed the fluorescent marker from an endogenous CP promoter [43].

The specificity of midgut expression in *Anopheles* female and male midguts directed from the CP promoter was in accordance with high throughput transcriptome data which indicates highly enriched CP gene transcription in midguts from both sexes [44]. In addition, the bias towards posterior midgut localisation and expression in late pupae closely reflects the endogenous pattern of CP expression [45]. The high levels of constitutive reporter gene activity observed in all lines probably reflects accumulation of Gal4 as the adult mosquito ages, as well as the amplification in signal provided by the Gal4-UAS system [30]. In comparison with the inducible trypsin promoter used in *An. stephensi* [12] which gave an approximate two fold increase after bloodfeeding, leading to only a 20 fold increase above wild type background, the CP promoter produced at least 50,000 fold greater activity in transheterozygous individuals than measured in the non-crossed responder lines.

No obvious increase in midgut fluorescence or luciferase activity was observed in all the lines examined following bloodfeeding. Previous research suggests that the *An. gambiae* CP gene is induced 4–8 fold within 3 hours of a bloodmeal, and then returns to unfed constitutive levels after 24–48 hours [45], [46]. More recent analysis has also demonstrated that CP transcription follows a pronounced daily rhythm and rises significantly between 4 hours either side of dusk, before gradually falling back to original levels after 24 hours [47]. Although, how this rhythm is modified following bloodfeeding was not determined. Transgenic studies that have utilized the *An. gambiae* CP promoter have indicated that transgene expression in the midgut followed the endogenous transcription pattern and was induced 3–6 hours after bloodfeeding [13], [31], [33]. From our transcriptional analysis it would appear that endogenous CP is also moderately upregulated following bloodfeeding in the line we have examined, and that Gal4 transcription from the CP promoter follows this expression profile closely. The lack of increase in luciferase gene transcription and enzyme activity, as well as eYFP intensity, indicates that increased levels of Gal4 mRNA are not converted into greater reporter gene activity in the time scale analysed. This would suggest that the Gal4-GFY and UAS combination is saturated at the constitutive level of expression provided by the CP promoter. At this point, it is not clear whether the UAS sites are saturated with Gal4 or if the transcriptional machinery involved with Gal4-GFY activation is already maximally active. Further development of the system will test other transactivators [30] to determine whether alternatives are less active or more responsive *in vivo*.

The large accumulation of transgene products produced from the CP-Gal4-GFY driver lines will prove useful in studies that examine very early stages of pathogen interaction in the midgut. Currently there are currently no promoters available that direct extensive expression prior to the formation of the peritrophic matrix (a protective chitinous layer produced early after feeding to surround the blood bolus) [43]. They will also prove valuable to study late stages of pathogen development in the midgut, since transgene expression is observed at least up to 14 days old. Similarly, it is now possible to target expression of other transgenes to the midgut whose function is not directly related to blood feeding, but nonetheless are important determinants of pathogen transmission. This would include genes such as those conferring insecticide resistance [10], altered redox potential [48] or those involved in membrane and lipid transport [49], [50].

All driver and responder combinations demonstrated an enrichment of female specific reporter gene expression, as would be expected from recent tissue specific transcriptomic data indicating that male mRNA signal is around 75% lower than in females 24 hours after a blood meal [44]. However, the magnitude of female enrichment observed in our analysis was dependant on driver line used. For example, progeny derived from line F crosses produced over 300 fold more specific activity in females compared to males. The majority of this difference originated from reduced relative male expression. It would thus appear that female expression is directed robustly from this CP promoter, whereas male expression is weaker and more subject to position effect. The use of gypsy insulator sequences in the driver constructs may alleviate this variation.

As has been reported recently during Gal4-UAS system development in *Aedes* [29], background expression was not visible outside the target tissue with a fluorescent reporter gene in transheterozygous *An. gambiae* individuals. However, using the more sensitive luciferase assay [51] available with this system in *Anopheles*, a very low level of activity was detected in carcass samples, which appeared to be almost entirely derived from non-Gal4 regulated expression from the UAS cassette insertions, since similar activities were detected in non-crossed responder lines. Similar levels of basal luciferase expression observed in more expansive *Drosophila* studies support the idea that it is unlikely that any UAS-transgenes are ever completely silent [42]. Many years of molecular analysis in *Drosophila* would also suggest that this limited basal activity is normally biologically inert and is most likely a consequence of non-specific polymerase II transcription throughout the genome [42]. This does not rule out the possibility that different UAS configurations or integration into different locations may reduce this activity.

In related work with *Tribolium*, consistent expression of Gal4 transactivators was only achieved by replacement of the core *Drosophila* heat shock promoter present on the original UAS constructs with a comparable endogenous sequence [28]. In contrast, this same core promoter is active in *An. gambiae* and is present on both the UAS regulated reporter genes, as well as the 3×P3 promoter used to express the selectable marker genes. More recent studies in *Aedes aegypti* have also demonstrated the utility of the core promoter to regulate Gal4-UAS dependant expression [29] in this related mosquito species. Thus, although we have no comparison with an endogenous core promoter, it would appear that the *Drosophila* sequence is sufficiently conserved to allow efficient expression of transgenes in mosquitoes.

As well as the initial validation of the Gal4 system in *An. gambiae*, we report the largest generation of independent transgenic *An. gambiae* lines to date. The work produced 21 transgenic lines, of which six carried intragenic transposon insertions that may have caused hypomorphic alleles. Similar frequencies of intragenic insertions have been achieved in other series of piggyBac mediated transformation of *An. gambiae* [52] which suggests that high throughput mutagenesis studies would be feasible if other aspects of transgenic mosquito selection, maintenance and preservation were optimised. Two lines, Wby and Xnt, which carry transgene insertions into orthologues of the *Drosophila Furin2* and *dachs* genes have been deposited with the Malaria Research and Reference Reagent Resource Center (MR4) to enable potential phenotypes from gene knock downs or hypomorphic alleles to be examined in the future. In addition, Wlm, displaying bright salivary gland expression, enabling easier dissection of this tissue has also been deposited in MR4.

In conclusion, the variant Gal4-UAS system described here functions well in *An. gambiae* to give robust and tissue specific transgene activation. Gal4 transactivation was evident in the progeny of all Gal4-UAS crosses, and appeared not to diminish in intensity over 20 generations. The Gal4-GFY driver used in this study gives intermediate levels of activation when used in conjunction with the UAS14i responder cassette in cell transfections. This Gal4 transactivator clearly works efficiently *in vivo* in combination with the CP promoter, however it may also be possible to produce different levels of target gene expression from the same promoter by utilizing other Gal4 transactivators described previously [30]. The availability of these variant Gal4 constructs with graded activation potential will also allow an informed choice of transactivator to match the potency of new driver promoters to be examined. The successful development of the Gal4-UAS system in *An. gambiae* is a major step forward for functional genetic characterization in this species. To facilitate distribution to the research community, these constructs and representative driver and responder lines will also be offered for general distribution to the MR4 malaria research facility. The establishment of driver and responder lines that direct expression in other tissues of importance in malaria transmission, insecticide resistance and host and mate seeking behaviour are obvious targets for future development.

## Methods

### Plasmid construction

#### CP-Gal4-GFY driver construct

PCR was used to amplify the 1.7 kb region upstream of the carboxypeptidase gene (CBPA1, AGAP009593), described by Edwards *et al* [45], using the G3 strain of *An. gambiae s.s.* DNA as template with NotI and EcoRV tagged primers. Primer sequences given in Table S1. This fragment was verified against the annotated sequence (VectorBase) and subcloned into pSL*LRIM1-Gal4-GFY [30] to replace the LRIM1 promoter. The entire CP-Gal4-GFY-SV40 sequence was then removed by AscI digest and cloned into pBac{3×P3-dsRed} [36].

#### Dual UAS-LUC-UAS-eYFP responder construct

The following cloning strategy was taken to construct a piggyBac vector carrying a UAS regulated luciferase and eYFP gene cassette flanked either side by gypsy insulator sequences. The gypsy insulator sequence from pH-Stinger (Drosophila Genomic Resource Centre) [53] was amplified by PCR with HindIII tagged primers (Table S1) and inserted 5′ to the UAS14i-LUC gene in pSL*UAS-LUC [30]. Orientation of the gypsy insert was checked by restriction digest, and the whole cassette subcloned into pBAC{3×P3-eCFPaf} [36], following AscI and FseI digestion, to give pB-gUAS-LUC. The gypsy insulator was again amplified by PCR but utilizing BamHI tagged primers, and this time subcloned 3′ of the UAS-LUC cassette to give pSL*UAS-LUCg. Separately, a nuclear localization tag was added to eYFP by fusion PCR, using plasmids eYFP-mem (Clontech) and pB*attB*[3×P3-dsRed2nls-SV40]*lox*66 [54] as templates respectively, and EcoRI and KpnI tagged external primers (Table S1). The eYFP-nls fusion product was then subcloned into pSL*UAS-LUCg to replace the luciferase gene. The UAS-eYFPnls-g cassette was then inserted into pB-gUAS-LUC using the FseI site (pB-gUAS-LUC-UAS-eYFPnls-g) and the orientation verified by diagnostic restriction digest (Figure 1B).

### Anopheline cell culture and transfection

The *An. gambiae* cell line Sua5.1, originally generated by Hans-Michael Muller [55], was kept in culture at 28°C in Schneiders medium (Invtrogen) supplemented with 10% fetal bovine serum (PAA) and 100 U/ml Penicillin and 100 µg/ml Streptomycin (Invitrogen). Cells were seeded on 24 well plates and cultured for 24 hours before transfection. Plasmid DNA was purified from bacterial cell culture using a Qiagen midi-prep kit according to manufacturer's instructions before ethanol precipitation and elution in ddH_2_0. Transfection was carried out using Qiagen Effectene reagent following the manufacturer's protocol with a ratio of 5 µl effectene and 1.6 µl enhancer to 300 ng DNA in 24 well plates. 100 ng of Gal4 driver plasmid (pSL-LRIM1-Gal4Δ [30]) was co-transfected with 100 ng of UAS responder plasmid (pSL*UAS-eYFPnls-g) and 100 ng of a pminAct5C-dsRed1 plasmid containing cytoplasmic dsRed1 under control of the Actin5C promoter [35]. Transfected cells were incubated at 28°C for at least 48 hours prior to microscope examination using fluorescein and rhodamine filters.

### Transformation of *Anopheles gambiae*

*Anopheles gambiae* mosquitoes (G3 strain) were reared according to the MR4 standard protocol. Early embryos were injected with a solution containing 350 ng/µl of the Gal4 driver plasmid (CP-Gal4-GFY) or the UAS responder plasmid (pB-gUAS-LUC-UAS-eYFPnls-g) and 150 ng/µl of the transposase helper plasmid, phspBac [56], following previous described methods [14]. Surviving larvae were then reared to adults and backcrossed to the parental strain. G1 progeny were screened for fluorescent eye marker using a Leitz MFLZIII microscope fitted with dsRed and CFP filter sets. Transgenic G1 larvae were pooled according to sex and cage of origin and backcrossed. Isofemale lines were obtained from individual female lays and the F2 progeny interbred. If possible selection for homozygous individuals was carried out at the F3 stage. The proportion of transgenic individuals in F2 and F3 generations for each line was counted and if this exceeded the number expected for a single transposon insertion, the line was discarded. In addition, six of the UAS lines were randomly sacrificed to facilitate maintenance, to leave six driver and nine responder lines.

### Determination of insertion location

Inverse PCR was carried out on each isofemale transgenic line to establish the location of the insertion event in the genome. DNA from at least 30 male and female adult mosquitoes was extracted using Qiagen GenomicTip 20/G columns as described in manufacturer's standard protocol. 2 µg of genomic DNA was then digested to completion with BfuCI (NEB), purified on diatomaceous earth [57] (Sigma) and 1 µg DNA then self-ligated at 5 ng/ml for at least 16 hours at 16°C using T4 DNA Ligase (NEB). Ligated DNA was then purified as above and resuspended in 30 µl of ddH_2_0. Genomic DNA flanking the piggyBac insertion sites was amplified by the PCR with primer pairs; left arm, ITRL1F and ITRL1R [15], and right arm, ITRR1F and ITRR1R (Table S1), using the manufacturer's recommendations for the Phire polymerase (NEB), and annealing temperatures of 65°C and 58°C for left arm and right arm reactions, respectively. Depending on the purity of the amplified products they were either sequenced directly from agarose gel extractions, or following reamplification from agarose plugs. Insertion sites were identified through BLAST [58] searches of VectorBase [59]. All genomic flanking sequences longer than the 200 bp limit on submission have been deposited in GenBank with accession numbers JN585644-JN585663.

### Gal4×UAS crosses

Crosses were performed on mosquito lines homozygous for the Gal4 (marked by dsRed) and UAS (marked by eCFP) transgenes. At least 10 females were used per cross with at least double the number of males. In total 20 different crosses were carried out with two experimental repeats for each cross. Larval progeny were screened to check expected inheritance of both red and cyan fluorescent eye marker proteins. Adults were examined at 3, 7 and 14 days after emergence. Dissections were carried out using a Leitz MFLZ III microscope using GFP-B, dsRed and CFP filters. Images were taken using a Zeiss Axiovert 200 M confocal microscope, an Olympus BX60 microscope fitted with a Nikon DSU2 camera, or through the dissecting microscope fitted with a Nikon P5100 digital camera.

### Luciferase assays

Seven day old mosquitoes were anesthetized with CO_2_ and dissections carried out in PBS. Midguts, carcasses, or whole mosquitoes were transferred to 200 µl of luciferase buffer [1× passive lysis buffer (Promega) supplemented with 1 µg/ml aprotinin (Sigma) and 1× EDTA-Free protease inhibitor cocktail (Roche). Samples from three mosquitoes were added per tube and the tube frozen in a dry ice ethanol bath before storage at −70°C. Three replicates for each cross were processed. Samples were later thawed on ice, homogenized for 1 min and then spun at full speed in a microfuge for 10 min at 4°C. The supernatant was kept at 4°C and used directly for luciferase and protein assays. Gal4-UAS midgut and whole mosquito samples were diluted 10 fold in luciferase buffer before luciferase assays were carried out. 1 mg/ml BSA was added to diluted midgut samples to prevent luciferase degradation. A Promega Luciferase Assay Kit (E1500) was used to assay luciferase concentrations in mosquito homogenates using a Lumat LB 9507 tube luminometer. Results were standardized to total protein concentration in samples using BioRad RC DC protein assay kits and measured using a VersaMax microplate reader (Molecular Devices) at 750 nm.

### Semi-quantitative RT-PCR

Mosquitoes were fed on human blood and midguts dissected at 3, 6, and 24 hours after feeding. Samples (N = 3, two replicates for each time point) were stored in 500 µl of Tri Reagent (Sigma). Unfed female mosquitoes (at 0 hours) were used as the reference time point. RNA was extracted according to Tri Reagent manufacturer's protocol and treated with Turbo DNase (Ambion). First strand cDNA was prepared from approximately 20 ng total RNA using Superscript III First-strand Synthesis System for RT-PCR (Invitrogen). Transcript abundance of the Gal4, luciferase and carboxypeptidase (AGAP009593) genes were examined by semi-quantitative RT-PCR using two sets of primers for each gene in order to monitor reliability of amplification (Table S2). cDNA levels were normalized against amplification of the *An. gambiae* ribosomal protein gene, rS60 (AGAP002122) [60]. PCR was carried out in a 25 µl reaction volume containing a final concentration of 1× PCR DreamTaq Green buffer (Fermentas), 0.1 mM dNTPs, 0.1 µM each primer (details in Table S2), 0.5 U DreamTaq Green DNA polymerase (Fermentas), and standardized cDNA quantities. Reaction conditions were 95°C for 3 min, followed by 22 to 32 cycles (see Table S2) of 95°C for 30 sec, 58°C for 30 sec, 72°C for 30 sec; followed by a final extension at 72°C for 10 min. Electrophoresis was carried on a TAE and agarose gel and products were visualized using ethidium bromide.

## Supporting Information

Figure S1 **Fluorescent marker protein expression in transheterozygous.** Images of three representative mosquitoes, U = UAS responder line, G = Gal4 driver line and GU = progeny of Gal4-UAS cross; (A) Brightfield, (B) DsRed filter, DsRed marker expression from the 3×P3 promoter in the Bolwig organ of the eyes and ganglia indicating G and GU larvae are positive for Gal4 driver. (C) CFP filter, eCFP expression in the same tissues of U and GU larvae. Note discernable lack of bleed-through between filter sets with red and blue markers.
(TIF)

Figure S2 **eCFP expression in the salivary glands of responder line Wlm.** (A) Ventral view of UAS responder line, Wlm, showing eCFP marker gene expression from the 3×P3 promoter in the Bolwig organ, ganglia, salivary glands and anal papillae. (B) Dorsal view of larvae showing eCFP expression in the Bolwig organ of the eyes, ganglia and salivary glands. (C) eCFP expression in dissected larval salivary glands. (D) eCFP expression in distal lateral lobes of adult salivary glands.
(TIF)

Figure S3 **Expression of fluorescent protein in the midgut.** Images A to F show a representative image of eYFP expression in the midgut of female progeny of crosses between driver lines Cln, Drt, Dgl, F, G and Ivr respectively with the responder line Wnd, photographed through GFP-B filter. All guts are from sugarfed mosquitoes.
(TIF)

Figure S4 **Expression of eYFP in male and female midguts of Gal4-UAS mosquitoes.** A representative image of eYFP expression in dissected midguts of a male (top) and female (bottom) heterozygous for the Gal4 and UAS cassettes under a GFP-B filter set for crosses involving the responder line, Mbl, and the driver lines Dgl and F (A and B respectively). All guts are from sugarfed mosquitoes.
(TIFF)

Table S1 **Primers used for plasmid construction and inverse PCR.** *The table describes the region amplified, the primer names and the primer sequences from 5′ to 3′. Bold font shows the non-homologous tag used for restriction enzyme digest and cloning.*
(DOCX)

Table S2 **Primers used for RT-PCR to determine transgene expression levels.** The table lists the target gene for amplification, the primer names, the primer sequences from 5′ to 3′ and the number of PCR cycles used to obtain suitable levels of amplification to enable semi-quantitative PCR.
(DOCX)
