# Supplementary figures and images for: Development of the Bi-Partite Gal4-UAS System in the African Malaria Mosquito, Anopheles gambiae
Source: PLoS One. 2012 Feb 13;7(2):e31552. doi: 10.1371/journal.pone.0031552 (PMC3278442; doi:10.1371/journal.pone.0031552)

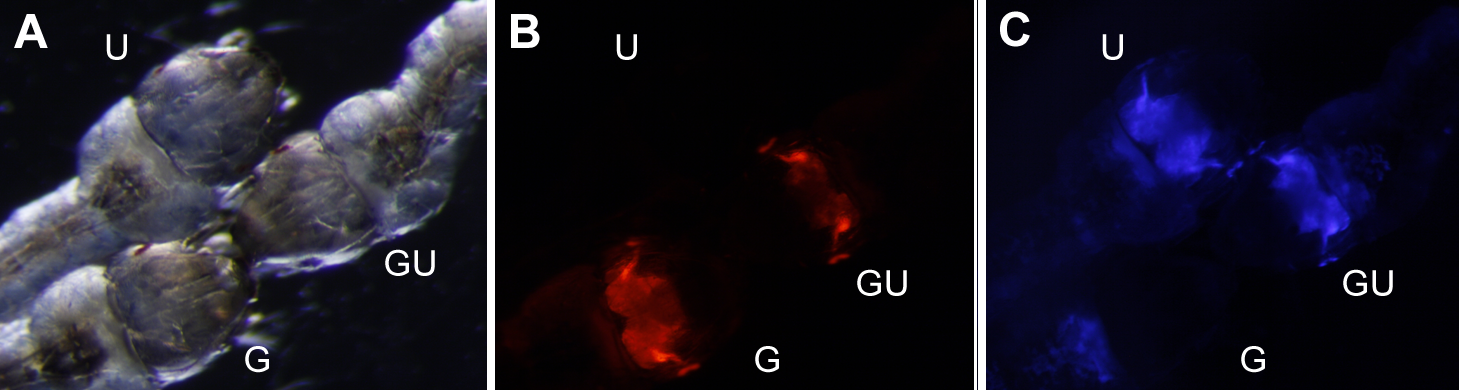

Supplement: Figure S1 — Fluorescent marker protein expression in transheterozygous. Images of three representative mosquitoes, U = UAS responder line, G = Gal4 driver line and GU = progeny of Gal4-UAS cross; (A) Brightfield, (B) DsRed filter, DsRed marker expression from the 3×P3 promoter in the Bolwig organ of the eyes and ganglia indicating G and GU larvae are positive for Gal4 driver. (C) CFP filter, eCFP expression in the same tissues of U and GU larvae. Note discernable lack of bleed-through between filter sets with red and blue markers. (TIF) [file pone.0031552.s001.tif]

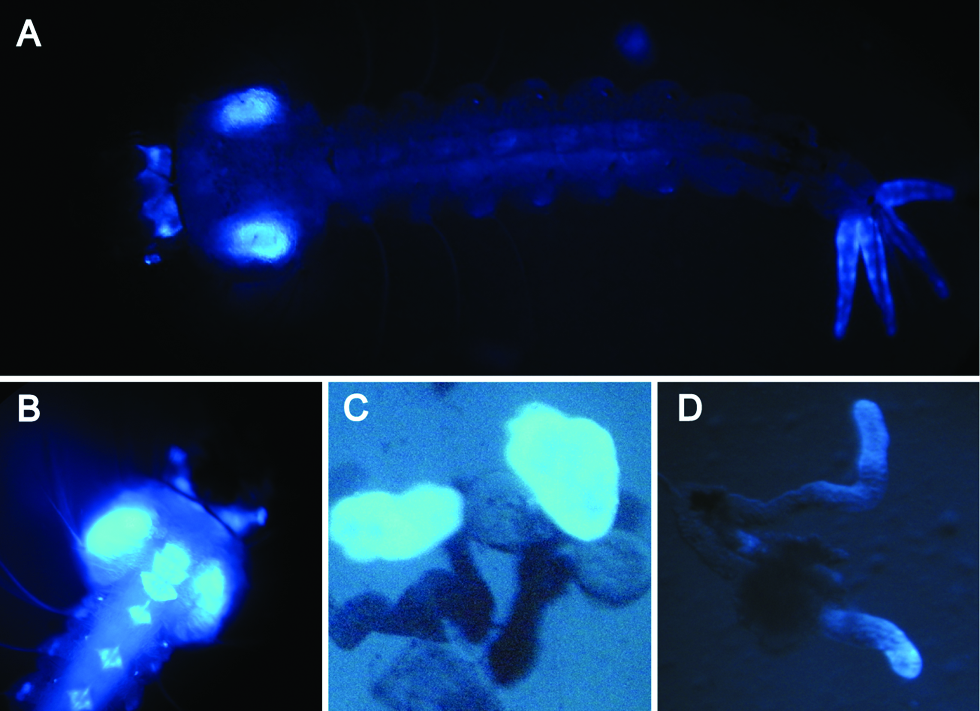

Supplement: Figure S2 — eCFP expression in the salivary glands of responder line Wlm. (A) Ventral view of UAS responder line, Wlm, showing eCFP marker gene expression from the 3×P3 promoter in the Bolwig organ, ganglia, salivary glands and anal papillae. (B) Dorsal view of larvae showing eCFP expression in the Bolwig organ of the eyes, ganglia and salivary glands. (C) eCFP expression in dissected larval salivary glands. (D) eCFP expression in distal lateral lobes of adult salivary glands. (TIF) [file pone.0031552.s002.tif]

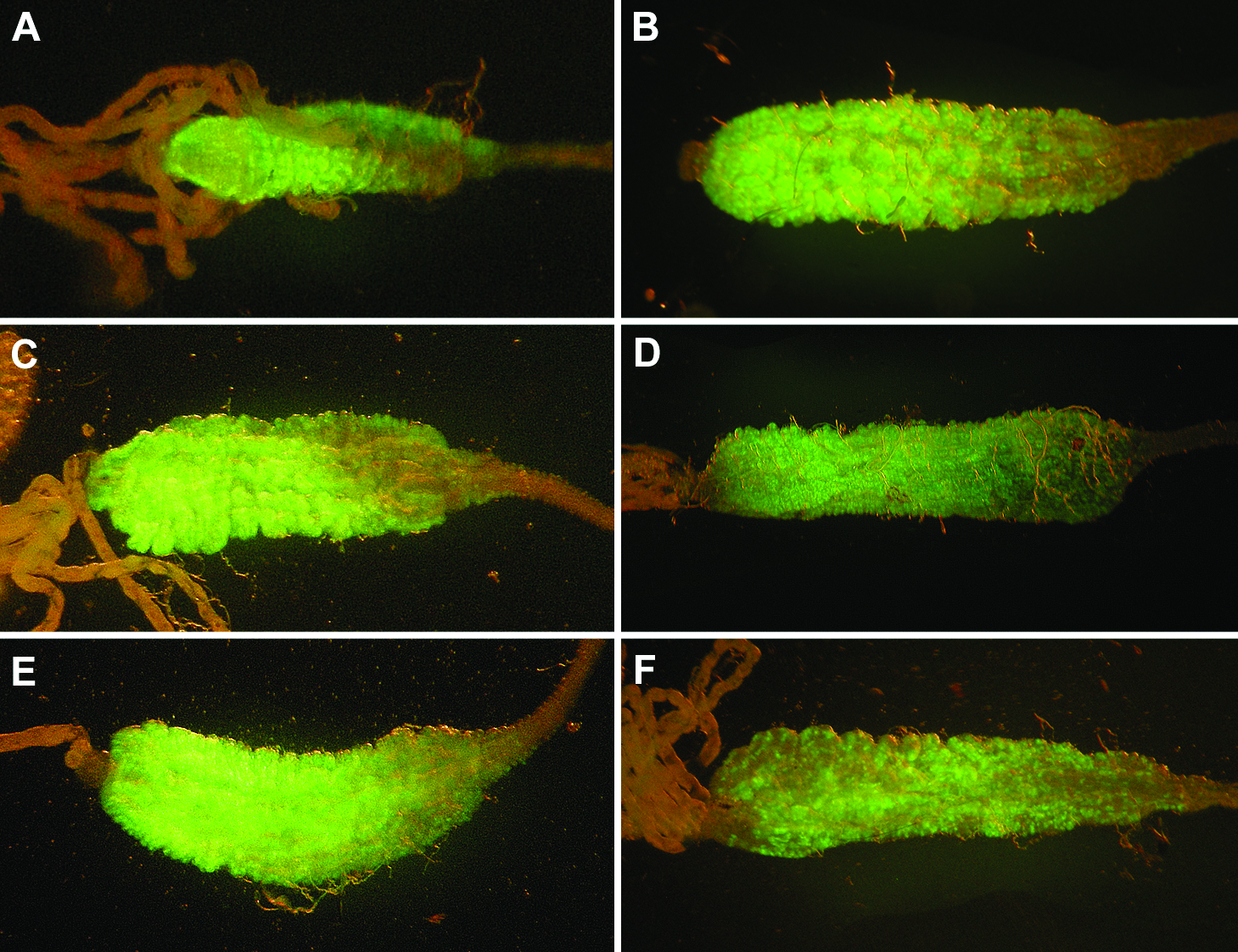

Supplement: Figure S3 — Expression of fluorescent protein in the midgut. Images A to F show a representative image of eYFP expression in the midgut of female progeny of crosses between driver lines Cln, Drt, Dgl, F, G and Ivr respectively with the responder line Wnd, photographed through GFP-B filter. All guts are from sugarfed mosquitoes. (TIF) [file pone.0031552.s003.tif]

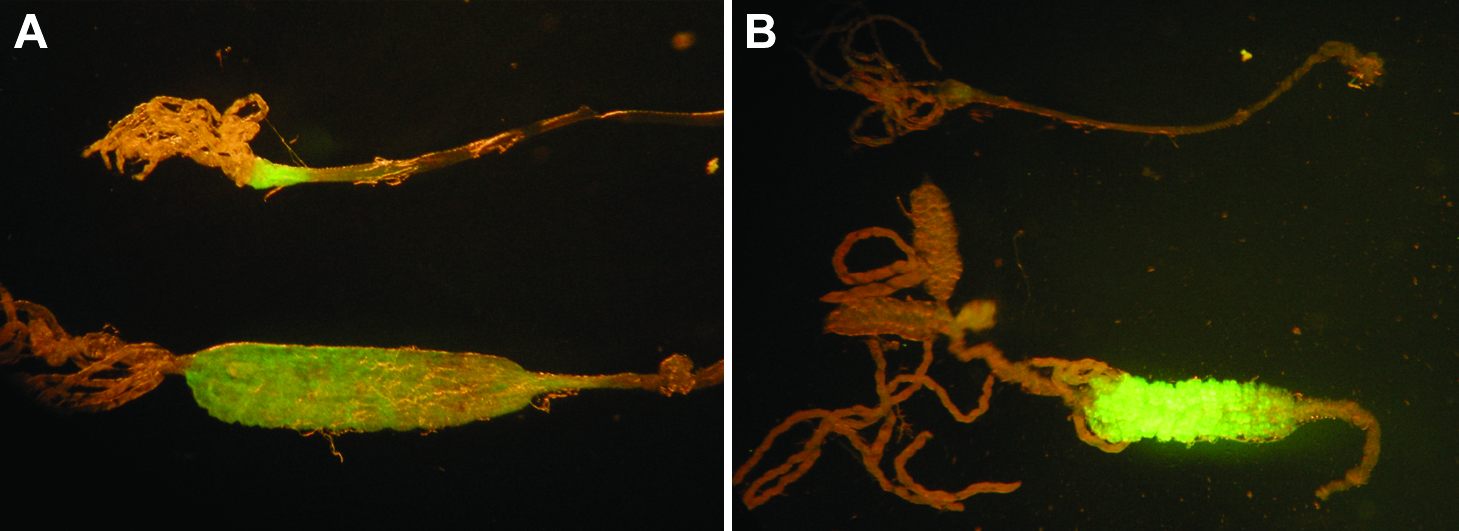

Supplement: Figure S4 — Expression of eYFP in male and female midguts of Gal4-UAS mosquitoes. A representative image of eYFP expression in dissected midguts of a male (top) and female (bottom) heterozygous for the Gal4 and UAS cassettes under a GFP-B filter set for crosses involving the responder line, Mbl, and the driver lines Dgl and F (A and B respectively). All guts are from sugarfed mosquitoes. (TIFF) [file pone.0031552.s004.tiff]
