# Supplementary material for: Development of the Bi-Partite Gal4-UAS System in the African Malaria Mosquito, Anopheles gambiae
Source: PLoS One. 2012 Feb 13;7(2):e31552. doi: 10.1371/journal.pone.0031552 (PMC3278442; doi:10.1371/journal.pone.0031552)
Supplement: Table S1 — Primers used for plasmid construction and inverse PCR. The table describes the region amplified, the primer names and the primer sequences from 5′ to 3′. Bold font shows the non-homologous tag used for restriction enzyme digest and cloning. (DOCX) [file pone.0031552.s005.docx]

| Amplicon | Primer Name | Details | Sequence 5’-3’ |
| --- | --- | --- | --- |
| Carboxypeptidase promoter | CP-Not-F  CP-Eco-R |  | **TTGTGCGGCCGC**CGCGTTGATGAAGAATGC  **TTGTCTATAG**GTTCAATCGACCGCTTGCT |
| Gypsy insulator | Gyp-Hind-F  Gyp-Hind-R |  | **TGTTAAGCTT**TGGTTTGTCCAAACTCATCAA  **TGTTAAGCTT**AGAATTGATCGGCTAAATGGT |
|  | Gyp-BmH-F  Gyp-BmH-R |  | **TGTTGGATCC**TGGTTTGTCCAAACTCATCAA  **TGTTGGATCC**AGAATTGATCGGCTAAATGGT |
| Fusion PCR for eYFP and NLS | eYFP-1F  eYFP-2R  eYFP-3F  eYFP-4R | External - EcoRI  Internal fusion  Internal fusion  External - KpnI | **TTGTGAATTC**CCAAAAGATCATGGTGAGCA  CTTGGGTCGAGATCTCTTGTACAGCTCGTC  GACGAGCTGTACAAGAGATCTCGACCCAAG  **TGTTGGTACC**GGATCTTACGGGTCCTCCAC |
