# Supplementary material for: Development of the Bi-Partite Gal4-UAS System in the African Malaria Mosquito, Anopheles gambiae
Source: PLoS One. 2012 Feb 13;7(2):e31552. doi: 10.1371/journal.pone.0031552 (PMC3278442; doi:10.1371/journal.pone.0031552)
Supplement: Table S2 — Primers used for RT-PCR to determine transgene expression levels. The table lists the target gene for amplification, the primer names, the primer sequences from 5′ to 3′ and the number of PCR cycles used to obtain suitable levels of amplification to enable semi-quantitative PCR. (DOCX) [file pone.0031552.s006.docx]

| Gene | Primer Name | Sequence 5’-3’ | No. cycles |
| --- | --- | --- | --- |
| Ribosome S60  AGAP002122 | rS60F  rS60R | GCCGAAGATTGTGAAGAAGC  CTTCGAACCGTAACCGATGT | 22 |
| Carboxypeptidase CP1 AGAP009593 | CP-1F  CP-1R | GTGCATACATTGCGTTCCAC  GCTTCGCCAGTGACTTTACC | 28 |
|  | CP-2F  CP-2R | GCGAGGATGCGAAGGTGCGT  TACGCGTACCCGTCCGGGTT | 30 |
| Gal4 | Gal-1F  Gal-1R | CCAAAGAAAAACCGAAGTGC  CCCTAGTCAGCGGAGACCTT | 32 |
|  | Gal-3F  Gal-3R | CATGCGATATTTGCCGACTT  AGCGGAGACCTTTTGGTTTT | 30 |
| Luciferase | Luc-1F  Luc-1R | ATCCATCTTGCTCCAACACC  TTTTCCGTCATCGTCTTTCC | 28 |
|  | Luc-2F  Luc-2R | AAGTGCGCTGCTGGTGCCAA  GAGAGGGGAGCGCCACCAGA | 22 |
